# Geographic Variation in Organ Size in a Toad (*Duttaphrynus melanostictus*)

**DOI:** 10.3390/ani13162645

**Published:** 2023-08-16

**Authors:** Weiye Deng, Long Jin, Duojing Qiu, Chengzhi Yan, Wenbo Liao

**Affiliations:** 1 Key Laboratory of Southwest China Wildlife Resources Conservation (Ministry of Education), China West Normal University, Nanchong 637009, China; 2 Key Laboratory of Artificial Propagation and Utilization in Anurans of Nanchong City, China West Normal University, Nanchong 637009, China; 3 Liziping Giant Panda’s Ecology and Conservation Observation and Research Station of Sichuan Province, Yaan 625407, China

**Keywords:** anurans, Hesse’s rule, digestion, altitude, latitude

## Abstract

**Simple Summary:**

The size of an organism’s organs not only relates to its body size and physiological function, but also offers important evidence of its adaptation to environmental changes. This study specifically investigated the geographical variation in organ size within the Asian common toad (*Duttaphrynus melanostictus*). Our findings revealed significant differences in the relative sizes of nine selected organs among various populations. Furthermore, we examined the effects of the geographical gradient on organ size, uncovering a positive correlation between the relative size of the testes and altitude and/or latitude.

**Abstract:**

Adaptive evolution is the process by which organisms change their morphological, physiological and biochemical characteristics to adapt to different environments during long-term natural selection. Especially, researching variation in organ size can provide important insights into morphological adaptation in amphibians. In this study, we comparatively studied differences in organ sizes (heart, lungs, liver, gallbladder, kidneys, spleen, digestive tract, testes and brain) among five geographical populations of the Asian common toad *Duttaphrynus melanostictus*. Our results revealed significant variations in the size of these nine specific organs among the populations. Notably, we observed a significant positive correlation between the relative size of the testes and latitude and/or altitude. However, no correlation was found between the relative size of the heart and the length of the digestive tract with altitude across populations, respectively, contradicting Hesse’s rule and the digestion theory. These findings suggest that our study does not provide substantial theoretical support for the adaptive evolution of organ size in this particular toad species, but rather contributes to the understanding of the evolution and adaptations of species’ different environmental conditions. Further research is warranted to delve deeper into the factors influencing organ size in amphibian populations.

## 1. Introduction

Phenotypic plasticity, the ability of organisms to modify their morphological and physiological characteristics in response to diverse environmental conditions, which is important for the adaptation of species to different environmental conditions, is widely recognized across animal taxa [1,2,3,4,5,6,7,8,9,10,11,12,13,14,15,16,17,18,19]. For example, the same genotype may exhibit distinct phenotypic traits as an adaptive mechanism in response to environmental fluctuations [20,21]. Organs with specific morphological and independent physiological functions in organisms also undergo adaptive changes in size and physiological functions in response to environmental influences [2,22,23,24]. Therefore, investigating variations in organ size and function becomes crucial in assessing the significance of phenotypic plasticity for the survival and fitness of organisms.

Energy storage plays a crucial role in influencing the geographic variation in organ size [2]. This function of energy storage becomes essential to ensure the survival and reproduction of organisms during periods of food scarcity [25,26,27]. In the case of amphibians, prolonged hibernation periods at high altitudes and/or latitudes necessitate greater energy accumulation in tissues such as the liver and body fat to support winter reproduction [28,29]. Notably, the liver and body fat mass of *Rana temporaria* exhibits an increase with rising latitudes [30]. Similarly, in *Bufo andrewsi*, liver size demonstrates a positive correlation with latitude [31], while the liver size of *Polypedates megacephalus* increases with altitude [8]. These findings provide compelling evidence supporting the adaptive evolution of organ size from an energy storage perspective.

Responses to environmental changes along latitudinal and altitudinal gradients are likely influenced not only by energy storage but also by factors such as food intake and the distribution of digested food components and oxygen within body organs. Hesse’s rule predicts that relative heart size increases with increasing altitude when oxygen supply and energy demands differ among environmental gradients [32]. Evidence for Hesse’s rule is provided by the fact that the size of the lungs and heart of *P. megacephalus* increase with altitude [8]. Additionally, the digestion theory predicts that foraging for large amounts of indigestible material results in a longer digestive tract. Indeed, *Rhinella spinulosus* displays a longer digestive tract in high altitudes [5], while the weight and length of the digestive tract in *Nidirana pleuraden* increases with increasing altitude [33].

The Asian common toad (*Duttaphrynus melanostictus*) is widely distributed in southern and southeastern Asia, occupying various habitats including forests, grasslands, cultivated lands and parks. This species grows and reproduces in various sites, including marshes, temporary ponds, puddles, and man-made ponds [34], rendering it an ideal candidate for studying the evolution of organ size and understanding adaptation to different environmental conditions and habitats. Therefore, in this study, we investigated patterns of geographic variation in the organ sizes of *D. melanostictus* among five populations. First, we examined the effect of the body condition on relative organ size. Next, we analyzed population-level variation in the size of nine selected organs (heart, lungs, liver, gallbladder, kidneys, spleen, digestive tract, testes, and brain) to explore whether organ sizes differed significantly across populations. Finally, we explored the relationship between organ size and altitude and/or latitude in different populations to verify whether Hesse’s rule and the digestion theory applied to this species, and we predicted that relative heart size and relative digestive tract size increased with increasing altitude or latitude in the species.

## 2. Materials and Methods

### 2.1. Study Sites

**Figure 1:**
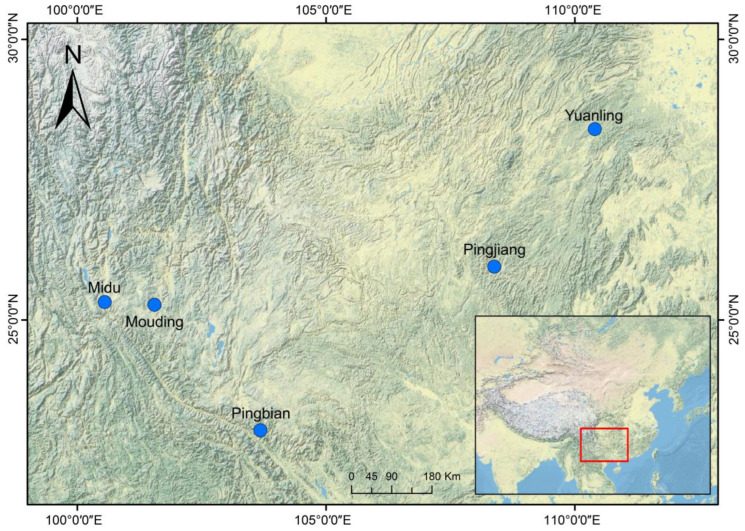
Map depicting the study sites for five *D. melanostictus* populations.

Field surveys and sample collection were carried out between April and August from 2018 to 2020. A total of five populations of *D. melanostictus* were collected from various locations, including Mouding, Midu, and Pingbian in Yunnan Province, Pingjiang in Guizhou Province, and Yuanling in Hunan Province (Figure 1). The study sites exhibited variations in altitude, average annual temperature, and average annual precipitation (Table 1).

### 2.2. Sample Collection

Upon reaching the designated survey site, we conducted daytime observations of the field environment, focusing on identifying egg bands, tadpoles, or newly metamorphosed individuals in the aquatic surroundings. During the night, using flashlight illumination, we randomly captured mature individuals (identified by their sexually mature characteristics such as song, nuptial pad, and pregnancy status) or subadults if the aforementioned characteristics were not evident. The captured individuals were then placed in a breathable cloth bag on site. To determine the sex of the collected individuals [35], we identified males by the presence of a brownish-black nuptial spur on the inner three fingers and their characteristic sound, described as “gu, gu”, when gently pressing the toad’s armpit from the back with a hand. In this study, a total of 153 specimens of *D. melanostictus* were collected.

### 2.3. Measurement of Body Size

After the toad was paralyzed by single-pithing, the body mass of the individual was weighed with a portable electronic balance, accurate to 0.01 g, and the body length (Snout-vent length, SVL) of the individuals was measured with an electronic vernier caliper, accurate to 0.01 mm [36,37]. The data measurements were performed by one person in the same standard and was recorded by another person to reduce the error. After completing the above operations, the individuals were preserved in a 4% phosphate buffered formalin for tissue fixation [38].

### 2.4. Anatomy and Measurement of Internal Organs and Reproductive Organs

After two weeks to two months of preservation, the fixed toads were removed, and the formaldehyde fixative was rinsed off the surface of the specimen under flowing water. The individuals were placed abdomen-up in a dissecting tray. We cut the skin and muscles on the surface along the mid-abdominal line with pointed surgical scissors. The organs (e.g., heart, gallbladder, lungs, liver, spleen, kidney and testes) of all individuals were taken out and loaded into the corresponding numbered microcentrifuge tubes. We then dried all organs in a thermostat at 60 °C for 24 h and then weighed the dried organs with an electronic balance accurate to 0.1 mg [39].

The connective tissue attached to the surface of the digestive tract was carefully removed with surgical scissors, and then the entire digestive tract (beginning of the esophagus to the vent) was removed; it was fixed on a foam board (in its natural curled state) with a pin, a straightedge was placed horizontally next to it as a scale and numbering information, and photographs were taken with a Motic Images 3.1 digital camera. Finally, the total length of the digestive tract was measured with the tpsDig2 software [8].

### 2.5. Anatomy and Measurement of Brain

The toad’s individual was placed in a dissecting tray with the backside up. All dissections, digital images and measurements were performed by Duojin Qiu. The brain and the dorsal medulla were removed completely and dried with absorbent paper. We placed them on a wax block and photographed on the dorsal, ventral, left and right sides using a Motic Images 3.1 digital camera on a Moticam2006 optical microscope. For dorsal and ventral views, we confirmed that the brain was horizontal and symmetrically positioned such that one hemisphere did not appear larger than the other. For paired structures, we measured only the width of the right hemisphere and doubled the volume. Photographs were imported into the tpsDig2 software, and the length (*L*), width (*W*), height (*H*) of the whole brain were measured, and the volumes of each brain region and the total brain were calculated using the following equation [40]:

(1) $$V=\frac{\left(L\times W\times H\right)\pi }{6\times 1.43}$$

### 2.6. Data Analysis

For the five populations, a total of 153 individuals’ data were taken from the heart, lungs, liver, gallbladder, kidneys, spleen, testes and digestive tract, and 147 individuals were used to obtain data on the brain. Since the Pingbian population had only one male sample, the testis-related analysis of this population was not counted.

All analyses were performed using the R software 4.2.3 [31]. Before analyses, continuous variables were log_10_-transformed to meet the normality assumption. The log-transformed values of these organ sizes (mass, volume, or length) were subtracted from the regression predicted values of log (organ size) relative to log (SVL) to obtain the residual of each organ, also known as organ relative sizes, which were also called organ coefficients and used in subsequent analyses. The body condition index of all individuals was obtained based on the regression predicted values of log (body mass) relative to log (SVL) [9].

By using linear regression analysis, we could explore the degree of influence of body condition index on the relative size of organs. For differences in organs among populations, we tested whether there were significant differences in relative organ sizes across populations by constructing a General Linear Model (GLM) with relative organ size as the dependent variable, body condition index as the control variable, and population as the independent variable. Furthermore, we used Linear Mixed Models (LMMs) with the organ relative size as the dependent variable, population as the random effect, and latitude and altitude as fixed effects to investigate whether there were significant differences in relative size organs across geographical gradients (altitude and/or latitude).

## 3. Results

### 3.1. Effect of Body Condition on Relative Organ Size

The mean values of heart, lung, gallbladder, liver, kidney, spleen, testes, digestive tract and brain for the five populations are reflected in Table 2. The effect of the body condition on each organ (heart, lungs, liver, gallbladder, kidneys, spleen, testes, digestive tract and brain) size was tested by linear regression analysis, which showed that the effect of the body condition on the relative size of the digestive tract and gallbladder was not significant (digestive tract: *t* = −0.293, *p* = 0.770; gallbladder: *t* = −0.353, *p* = 0.725; Table 3), while the effect of body condition on the other seven organ relative sizes were significantly affected (*p* < 0.05; Table 3).

### 3.2. Population Variation and Geographic Variation in Organ Size

As depicted earlier, a significant linear relationship was observed between the relative sizes of the organs and body condition, with the exception of the digestive tract and gallbladder. Therefore, differences in relative size of organs among populations were analyzed using the general linear model when correcting body condition effects.

The GLM showed that the relative size of the nine specific organs differed significantly among populations (all *p* < 0.05; Table 4). 

**Figure 2:**
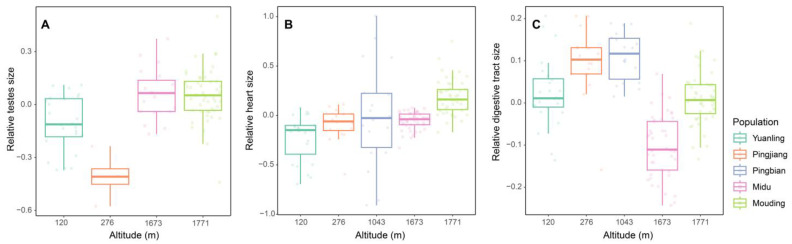
The relationships between altitude and relative testes size (**A**), heart size (**B**), and digestive tract size (**C**) among *D. melanostictus* populations.

**Figure 3:**
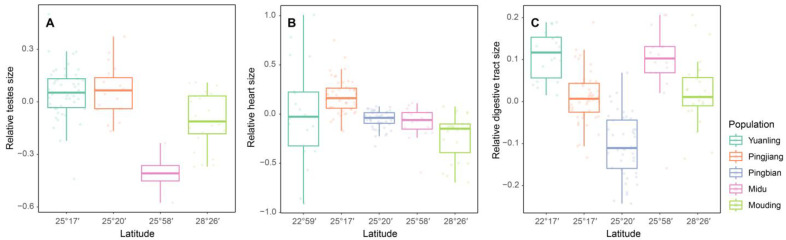
The relationship between latitude and relative testes size (**A**), heart size (**B**), and digestive tract size (**C**) among *D. melanostictus* populations.

The results of the LMMs analysis revealed a significant positive correlation between the relative size of testes and altitude and/or latitude (altitude: *t* = 5.624, *p* < 0.001; latitude: *t* = 4.551, *p* < 0.001; Table 5; Figure 2A; Figure 3A). However, we did not find significant relationships between heart size and altitude and/or latitude (Figure 2B; Figure 3B) and between digestive tract and altitude and/or latitude (Figure 2C; Figure 3C).

## 4. Discussion

Our findings have shown that the relative size of testes increased with a rising altitude and/or latitude. We first observed significant differences in the relative sizes of the nine representative organs (heart, lungs, liver, gallbladder, kidneys, spleen, digestive tract, testes, and brain) among the geographical populations. Consequently, we conducted further analyses to explore the relationship between relative organ size and altitude and/or latitude. While we observed a significant and positive correlation between the relative size of testes and altitude and/or latitude, the heart and digestive tract did not exhibit increases with rising altitude and/or latitude. These observations were inconsistent with both Hesse’s rule and digestion theory. 

According to life-history theory, trade-offs between current reproductive investment and future reproductive success and/or survival play a crucial role in organisms’ adaptation to environmental changes. This trade-off was evident in testis size, which serves as the primary measure of male reproductive investment and is influenced by varying intensity of sexual selection [41]. Notably, several North American bird species display a positive correlation between testes size and latitude, attributable to the higher sexual selection pressures experienced at higher latitudes [41]. In amphibians, previous studies have demonstrated a positive correlation between the relative testes size of *P. megacephalus* and *Fejervarya limnocharis* with altitude [25,42], while *B. andrewsi* showed no significant altitudinal or latitudinal differences in testes size [43]. In this study, we found a significant and positive correlation between the relative testes size and altitude and/or latitude in *D. melanostictus*, supporting the predictions of life-history theory. This correlation suggests that species at higher altitudes and/or latitudes are subject to robust sexual selection and exhibit larger relative testes sizes. This observation further emphasizes the relevance of life-history theory in understanding the adaptive mechanisms of organisms to environmental changes. 

Variation in relative liver size is associated with altitude and/or altitude because energy stores are necessary to ensure the survival and reproduction in organisms [25,26,27]. The amount of energy reserves is associated with environmental conditions [26,44]. For instance, individuals living at high altitudes and/or latitudes are exposed to environmental stresses such as low oxygen, lower and variable temperatures, and high UV radiation should have increased energy requirements and intakes [45]. As the main energy storage organ in amphibians, the liver plays an important role in adapting to environmental changes. Previous studies have shown that the liver size increases with increasing latitude in *R. temporaria* [30] and altitude in *P. megacephalus* and *B. andrewsi* [8,31]. However, Zhao et al. [46] found that individuals at high altitudes and/or latitudes do not show larger livers in *B. andrewsi*. In this study, we also found a non-significant correlation between relative liver size and altitude and/or latitude. These findings highlight the complex and varied responses of liver size to environmental conditions in different amphibian populations.

Animals living at high altitudes and/or latitudes are often stressed by both low temperature and low oxygen conditions, thus evolving larger hearts and lungs to meet normal metabolic demands to take in more oxygen [8,47]. Meanwhile, animals may be limited in aerobic activities, such as exercise and thermogenesis, to adapt to the low oxygen conditions at high altitudes and/or latitudes [45,48]. Previous studies have shown that consistent with Hesse’s rule, vertebrates (i.e., *Peromyscus maniculatus sonoriensis* and *P. megacephalus*) at high altitudes have larger hearts and lungs than those at lower altitudes [2,8], which may result from the lower partial pressure of oxygen at higher altitudes. However, a previous study did not find a positive correlation between heart and lungs and altitude and/or latitude in *B. andrewsi* [46]. We similarly did not find a correlation between the relative size of heart and lungs and both altitude and/or latitude, suggesting that oxygen supply pressure might not play an important role in shaping changes in the relative size of the heart and lungs in *D. melanostictus*, which do not support Hesse’s rule. 

There is evidence that both thermostatic and ectothermic animals are able to adapt digestion-related traits to changes in environments [5,49,50,51]. Indeed, individuals compensate for lower-quality food by altering digestive mechanisms such as accelerating the turnaround time of high-fiber foods, increasing the capacity of the intestine, and enhancing the absorption of nutrients in the small intestine [33,52,53]. A previous study has shown that the digestive tract weight and length of *P. pleuraden* increase with increasing altitude, likely because under harsh environmental conditions at high altitudes, *P. pleuraden* is forced to accept some indigestible (low-quality) food [33]. In contrast, the digestive tract of *B. spinulosus* became shorter with increasing altitude due to the availability of more easily digestible animal foods [5]. In our study, however, we did not find a significant correlation between relative digestive tract length and altitude and/or latitude, which does not align with the predictions of the digestion theory. These findings suggested that factors other than altitude and/or latitude might influence the variation in the relative size of the digestive tract in *D. melanostictus*.

Brain size exhibits significant variations at both interspecific and intraspecific levels in animals [54,55,56]. Two main hypotheses have been proposed to explain brain-size variation, most of which regard a relationship to selective benefits due to cognitive demands [57,58,59]. The cognitive-buffer hypothesis (CBH) predicts that an advantage of a relatively larger brain is to increase cognitive abilities in unstable environments [60]. For instance, artificial selection on large-brained individuals evidently enhances learning ability, leading to success in such things as building food caches among birds [61], numerical abilities in guppies [62], and problem solving in mammals [63]. However, the CBH cannot explain trade-offs between developmental costs and benefits of larger brains [64]. Larger brains need either an increase in total metabolism or a reduction in allocation of energy to other organs [65,66]. In contrast to the CBH, the expensive-brain framework hypothesis (EBF) states that a relatively smaller brain results when animals experience energy shortages in fluctuating environments [67]. Indeed, periodic food scarcity is expected to constrain brain-size evolution in frogs [40]. 

Larger brain size in organisms evolved to adapt to new or changing environmental conditions because the relatively larger brain size facilitates enhanced cognitive abilities and behavioral plasticity of individuals, thus helping them to better avoid natural predators, find food, and adjust reproductive behavior [68,69,70,71,72,73,74,75,76,77,78,79,80,81]. Previous studies have found that the relative brain size of birds and mammals increases in response to new environments [68,71]. However, environmental seasonality hampers brain size when temporary food shortage is buffered by stored energy [82]. For example, there is evidence that absolute and relative brain volumes are significantly and positively correlated with environmental seasonality among ten *B. andrewsi* populations [40]. Nonetheless, two species (i.e., *F. limnocharis, Hylarana guentheri*) do not display correlations between absolute and relative brain size and environmental seasonality across populations [83,84]. In the present study, the relative brain volume of *D. melanostictus* did not significantly correlate with altitude and/or latitude possibly because of the small sample size of the populations.

## 5. Conclusions

In conclusion, our study revealed significant and positive correlations between the relative size of testes and both altitude and latitude, supporting the predictions of the life-history theory. However, we did not find any significant correlations between the relative size of heart, lungs, liver, gallbladder, kidneys, spleen, digestive tract, and brain in *D. melanostictus* and altitude and/or latitude across populations. This suggests that heart size variation of *D. melanostictus* cannot be explained by Hesse’s rule, and the changes in the relative size of the digestive tract do not align with the predictions of the digestion theory. It is essential to consider the potential effects of climate change, habitat alteration or invasion of new habitats, and phylogenetic relationships among populations on variations in organ size, as these factors can also influence the adaptive abilities of animals. Future studies should take these factors into account to gain a comprehensive understanding of the determinants of organ size variations.

## Figures and Tables

**Table 1:** Location of *D. melanostictus* for five populations in China.

Study Site	Sample Size	Longitude (E)	Latitude (N)	Altitude (m)	Average Annual Temperature (°C)	Average Annual Precipitation (mm)
Midu	45	100°33′	25°20′	1673	17.3	824.4
Mouding	50	101°33′	25°17′	1771	15.8	872
Pingjiang	16	108°23′	25°58′	276	18.0	1300
Pingbian	18	103°41′	22°59′	1043	16.5	1650
Yuanling	24	110°24′	28°26′	120	16.7	1400

**Table 2:** Descriptive information about the study sites, snout-vent length (SVL), body mass and organ size with mean ± SE in *D. melanostictus*.

Factors	Midu	Mouding	Pingjiang	Pingbian	Yuanling	Total
SVL (mm)	62.79 ± 8.77	61.30 ± 4.25	53.08 ± 8.87	71.42 ± 21.15	51.11 ± 2.64	59.25 ± 16.69
Weight (g)	31.07 ± 12.79	24.87 ± 5.33	18.55 ± 8.86	42.18 ± 30.02	12.87 ± 2.59	24.55 ± 23.77
Digestive tract (mm)	87.17 ± 18.35	110.81 ± 17.82	123.09 ± 29.70	155.46 ± 37.98	95.41 ± 14.97	122.97 ± 39.31
Heart (mg)	26.25 ± 10.70	42.86 ± 21.54	16.92 ± 7.61	51.83 ± 47.26	10.97 ± 4.94	28.19 ± 36.01
Lungs (mg)	46.99 ± 22.79	42.00 ± 18.38	25.66 ± 13.89	97.44 ± 94.37	32.10 ± 30.34	55.70 ± 71.68
Gallbladder (mg)	2.06 ± 1.13	1.69 ± 0.79	1.26 ± 0.52	3.03 ± 1.97	1.89 ± 0.73	2.34 ± 1.59
Liver (mg)	163.67 ± 78.76	125.09 ± 47.84	132.96 ± 67.44	416.79 ± 400.08	50.29 ± 38.96	206.72 ± 309.33
Kidneys (mg)	29.06 ± 14.45	32.20 ± 15.62	23.01 ± 10.92	54.55 ± 42.77	15.26 ± 7.57	31.74 ± 33.58
Spleen (mg)	5.69 ± 4.41	2.10 ± 1.67	2.44 ± 1.62	3.19 ± 2.37	1.77 ± 0.57	2.38 ± 1.77
Testes (mg)	16.61 ± 5.61	12.12 ± 3.95	4.72 ± 3.08	7.30	3.64 ± 1.30	3.84 ± 1.41
Brain (mm^3^)	84.04 ± 14.04	60.25 ± 10.64	90.51 ± 153.56	84.41 ± 21.75	48.41 ± 3.82	63.48 ± 23.85

**Table 3:** Regression analysis of body condition index and relative organ sizes in *D. melanostictus*.

Organ	β	t	P	R^2^
Digestive tract	−0.030	−0.293	0.770	0.001
Heart	0.827	2.999	0.003	0.057
Lungs	0.915	3.611	<0.001	0.081
Gallbladder	−0.107	−0.353	0.725	0.001
Liver	1.160	4.016	<0.001	0.099
Kidneys	0.786	3.426	<0.001	0.073
Spleen	1.829	4.821	<0.001	0.161
Testes	0.593	2.306	0.023	0.053
Brain	0.494	6.409	<0.001	0.222

**Table 4:** The organ size variation among populations in *D. melanostictus* when correcting for the body condition index using the General Linear Model (GLM).

Factor	Digestive Tract	Heart	Lungs	Gallbladder	Liver	Kidneys	Spleen	Testes	Brain
Population									
F	47.218	14.983	2.656	2.832	10.199	3.396	16.096	17.210	41.142
P	<0.001	<0.001	0.035	0.028	<0.001	0.011	<0.001	<0.001	<0.001
Body condition index									
F	17.120	9.7775	13.263	0.053	8.609	12.118	9.468	1.974	21.718
P	<0.001	0.002	<0.001	0.819	0.004	<0.001	0.003	0.163	<0.001

**Table 5:** The effects of altitude, latitude, and population on variation in organ size among populations of *D. melanostictus* when correcting for body condition index using Linear Mixed Models (LMMs).

Source	Random		Fixed				
	VAR	SD	Estimate	SE	df	*t*	*p*
Digestive tract							
Population	0.003	0.059					
Residual	0.005	0.071					
Latitude			−2.942	1.345	2.125	−2.187	0.153
Altitude			−0.197	0.085	2.064	−2.329	0.141
Heart							
Population	0.014	0.119					
Residual	0.055	0.235					
Latitude			−0.027	2.844	2.341	−0.010	0.993
Altitude			0.238	0.177	2.174	1.341	0.303
Lungs							
Population	<0.001	<0.001					
Residual	0.062	0.249					
Latitude			−1.548	1.183	146.000	−1.308	0.193
Altitude			0.054	0.066	146.000	0.831	0.407
Gallbladder							
Population	0.009	0.096					
Residual	0.044	0.210					
Latitude			−1.886	2.449	1.695	−0.770	0.534
Altitude			−0.019	0.150	1.475	−0.126	0.914
Liver							
Population	0.011	0.105					
Residual	0.069	0.263					
Latitude			−5.186	2.630	1.882	−1.972	0.195
Altitude			−0.047	0.162	1.688	−0.289	0.804
Kidneys							
Population	0.001	0.033					
Residual	0.049	0.222					
Latitude			−1.912	1.282	3.761	−1.491	0.215
Altitude			0.024	0.075	2.634	0.317	0.775
Spleen							
Population	0.044	0.209					
Residual	0.067	0.260					
Latitude			2.147	4.868	2.297	0.441	0.697
Altitude			0.180	0.304	2.150	0.594	0.609
Testes							
Population	<0.001	<0.001					
Residual	0.022	0.148					
Latitude			14.907	3.276	93.000	4.551	<0.001
Altitude			0.788	0.140	93.000	5.624	<0.001
Brain							
Population	0.005	0.069					
Residual	0.004	0.061					
Latitude			−0.180	1.545	2.084	−0.117	0.917
Altitude			0.056	0.098	2.046	0.575	0.622

## Data Availability

The data presented in this study are available in the article.
